# The role of podiatry in the early identification and prevention of lower limb venous disease: an ethnographic study

**DOI:** 10.1186/s13047-022-00588-7

**Published:** 2022-11-30

**Authors:** Peter James Joseph Roberts, Karen Ousey, Caroline Barker, Sarah Reel

**Affiliations:** The University of Huddersfield, Queensgate, Huddersfield, HD1 3DH England

**Keywords:** Podiatry, Venous insufficiency, Venous disease, Lower limb venous disease, Early identification, Prevention

## Abstract

**Background:**

Lower limb venous disease can cause significant pain, loss of mobility, and can be detrimental to an individual’s quality of life. Manifestations of venous disease often pose a substantially negative impact on patients and place a high demand on finite healthcare resources. Whilst this problem is internationally recognised, most research and discourse has predominantly focussed on treatment of leg ulceration and prevention of recurrence. Prevention of lower limb venous disease progression to the first ulceration has received far less attention. Overall, the care of this condition appears to rest in the domain of medicine and nursing yet podiatry, a profession with responsibility for the lower limb and foot, is conspicuous by its absence from the literature.

**Methods:**

An ethnographic approach was used to gather data from 26 participants through observation, semi-structured interviews, and a focus group interview. Qualitative analysis was conducted using the framework approach.

**Results:**

The findings revealed an identity crisis within the podiatry profession. Evidence emerged of ritual and routine practices that did not include lower limb venous disease. External control over practice limited the professional autonomy of podiatrists determining their own activities. Inter-professional relationships with nursing, and perceptions of boundaries that venous disease was a nursing role were also found to be limiting factors.

**Conclusions:**

This research revealed that podiatry does not occupy a substantive role in contributing to the early identification and prevention of lower limb venous disease. Policy, education, research and practice changes are all required to enhance the contribution of podiatry to reduce the burden of this disease.

## Background

Lower limb venous disease can be painful and detrimental to mobility, self-esteem and the overall quality of life of individuals with venous leg ulcers having a negative emotional impact and causing debilitating discomfort [1–3]. Significantly, quality of life research reveals lower limb venous disease is comparable with higher profile conditions such as myocardial infarction, cardiac failure, and obstructive pulmonary disease [4–6]. Global data suggest high prevalence of Lower Limb Venous Disease [7, 8], for example the vein consult program [9] held across Eastern Europe, Western Europe, South and Latin America, and the Middle and Far East, found 61.2% of 69,866 patients had signs of lower limb venous disease when screened opportunistically when attending for non-venous related matters. Studies in Germany, Italy and the UK have also revealed prevalence ranging from 7% to 15.8% of people with more advanced stages involving oedema, skin changes and ulceration [10–12]. Data from community healthcare in England [13, 14] suggests that venous leg ulceration constitutes one of the highest prevalence and associated costs to health service resources. It is without doubt a burdensome and significant disease.

Expert opinion has suggested primary prevention of venous leg ulceration requires a clear emphasis in healthcare practice [15, 1, 16–25]. These papers are predominantly written from a nursing perspective and indicate a gap in respect of other professional groups. Podiatry, as a profession responsible for the leg and foot, is conspicuous by its absence from the collective lower limb venous disease literature. Podiatry has a focus on the lower limb and foot and reputedly podiatrists have time to engage patients in preventative healthcare discussions [26, 27].

There are 13,031 podiatrists registered with the Health and Care Professions Council (HCPC) in the UK [28]. Farndon et al. defined core podiatry as: “treatment of the nails, corns and callus and also giving footwear and foot health advice” ([29]- p.7). However, podiatrists also contribute significantly to the care of people with diabetes mellitus, peripheral arterial disease, musculoskeletal conditions, neurological conditions and rheumatology [30]. For these conditions, they have established significant roles in early identification, prevention and management [31–33]. Similarly, promoting public health agendas is becoming embedded in podiatry where work to support smoking cessation and falls prevention, for example, has supplemented core clinical activities [34, 35]. In these terms, podiatry is concerned more with the lower limb and whole person than it is isolated to the foot. Furthermore, the regulatory standards of proficiency state that podiatrists should: “Know and be able to interpret the signs and symptoms of systemic disorders as they manifest in the lower limb and foot…” ([36]-pg. 12). Due to this expertise in preventative medicine and observation of the lower limb, and the burden of lower limb venous disease, it is necessary to question what role the profession takes in early identification and prevention of such.

Podiatry has pursued a ‘professional project;’ a process of developing a unique set of skills to control the market for that expertise [37, 38]. The professional project of podiatry has included recognition for independent and supplementary prescribing rights; the development of podiatric surgery, and growth of named specialist roles in areas such as diabetes [39–41]. Professionalisation has been essential in distancing podiatry from connotations of the term chiropody, to enhance the perception of podiatry as having higher order skills and a more in-depth curative scope of practice [40]. However, podiatrists continue to register with the HCPC under the dual protected titles chiropodist/podiatrist [36]. This results in confusion amongst the public and other health professionals and is a cause for concern amongst podiatrists due to the low status chiropody implies [42]. Through the process of professionalisation, podiatrists are attempting to gain independence and autonomy with a view to being recognised as lower-limb and not simply foot experts. This last point was a particular recommendation of the Saks report in 2021 investigating the future of the podiatry profession and the strategy for podiatry workforce and education development [43]. Prevention of lower limb venous disease deterioration certainly sits within the bracket of extending practice beyond the foot and it would appear to be a perfect match of need, opportunity and expertise for podiatrists to be more overtly involved.

At the time of writing the role of podiatrists in the early identification and prevention of lower limb venous disease is unknown. According to Farndon [30] most patients treated by podiatrists in the UK are aged 62 years and over, significantly this is the age group most at risk of developing lower limb venous disease [7]. A critical exploration of the role these 13,031 podiatrists play in lower limb venous disease prevention is overdue, as care of people with lower limb venous disease appears to be solely in the domain of registered nurses and general practitioners (GPs) [44]. Despite publication of evidence-based guidelines and care pathways [45, 46] there is disparity nationally regarding pathways and multidisciplinary care at all levels of the disease [44]. Consequently, with GPs and registered nurses facing mounting strain and staff shortfall [47], other professional groups must recognise and use opportunities to contribute time and expertise to combat the growing burden of the disease and its complications. NHS England [27] suggests Allied Health Professionals (AHPs) are in a prime position for public health promotion and preventative care. Podiatry is one such profession and given its proximity to the lower limb is conspicuous by its absence in lower limb venous disease discourse.

### Methodology

An ethnographic methodology was used to explore the role of podiatrists in the North of England in the early identification and prevention of lower limb venous disease. Ethnography is a qualitative approach which situates the researcher within a culture to enable description and explanation of activities and behaviours [48]. Multiple data collection methods are used including observations, interviews and focus groups to enable triangulation of findings. Ethnography is a technique increasingly used for understanding health care and health services and to develop knowledge on topics about which little has already been published [48] and was therefore an appropriate approach for this research where little was known about podiatry’s current role.

### Ethics

Ethical approval was gained from the University of Huddersfield, School of Human and Health Sciences Research Ethics Panel, and the NHS Integrated Research Application System (IRAS). Reference numbers:—Research Ethics Council (REC) reference: 15/NW/0137; IRAS project ID: 124,438. Informed consent was obtained from all participants and data were anonymised and kept securely according to General Data Protection Regulations and the data storage regulations of the host institution. Participants were given a pseudonym for the purposes of analysis and publication. Patient views and personal data were not included in this study but permission was obtained for the researcher to observe their treatment appointment. Participants were also reassured that they were free to withdraw from the study until the date that formal analysis commenced.

### Sampling, recruitment, and data collection

Data were collected in three stages. In the first stage, 10 podiatrists were observed in clinical practice for a minimum of one day. Six practitioners worked in private practice and 4 within the National Health Service. Semi-structured interviews were undertaken with the ten participants following a period of observation. Recruitment was made via e-mail responses to adverts sent out by either NHS podiatry managers or directly to private practice e-mail addresses.

For the second stage, 8 semi-structured interviews were undertaken with podiatry academics, National Health Service podiatry service managers and pre-practice podiatry graduates. A piloted interview guide was used to maintain focus whilst encouraging participants to have their own voice.

The final stage involved an inter-professional focus group interview with:- podiatrists; National Health Service and private practice podiatry managers; specialist high-risk podiatrists; district nurses and a vascular nurse specialist. The group was asked to discuss the role of podiatry in the early identification and prevention of lower limb venous disease. Minimal prompts were required as the members explored the topic in an in-depth and enthusiastic discussion.

The sampling strategy was purposive within a single geographical area with participants recruited from both private and NHS podiatrists with a range of experience levels and fields of practice (Tables 1, 2 and 3). The sample size was predominantly guided by the recruitment of a broad range of participants who could illuminate the current practice within the chosen geographical location. Data saturation was also used as a guide to sample size. The authors believe saturation was achieved as the point was reached where themes were being repeated with no new concepts emerging. The total sample was:—participants observed and interviewed (*n* = 10), participants interviewed only (*n* = 8), participants in the focus group interview (*n* = 8).

**Table 1:** Sample for the observations with interviews

**Participant Pseudonym and highest qualification**	**Age**	**Years registered as a podiatrist**	**Current practice**
Alice (BSc)	55	20	100% private practice from 2001 – General podiatry
Beryl (BSc)	40	19	50% private: 50% NHS – General podiatry and musculoskeletal speciality
Cathy (BSc)	52	31	100% private practice between 2 shared clinics – General podiatry
Donna (BSc)	39	18	40% Private: 60% NHS – General podiatry and diabetes speciality
Eddie (BSc)	52	23	60% private: 40% NHS – general podiatry and musculoskeletal speciality
Fran (MSc)	37	16	20% private practice: 40% any qualified provider (AQP) podiatry service: 40% NHS management. General podiatry and high-risk podiatry speciality and research
Georgina (BSc)	31	10	100% NHS – general podiatry
Heather (BSc)	55	34	100% NHS – general podiatry
India (BSc)	48	27	100% NHS – new patient assessments and health promotion speciality
James (BSc)	28	7	100% NHS – general podiatry and musculoskeletal speciality

**Table 2:** Sample for the individual interviews

**Participant Pseudonym**	**Age**	**Years registered as Chiropodist/podiatrist**	**Current practice**
Kate	50	25 (hands on practice) 5 years 100% management	100% NHS podiatry management. Clinical background in paediatric biomechanics
Leonard (MSc, PhD)	57	36	70% NHS management 15% academic role 15% other consultancy work and research
Martin (MA)	Un-disclosed	Un- disclosed	100% academic management and lecturing with a history of high risk and general podiatry
Naomi	22	Pre-practice	
Olivia	25	Pre-practice	
Paul	42	Pre-practice	
Queenie	35	Pre-practice	
Rachael	33	5	100% podiatry lecturing after 3 years 100% NHS practice – general podiatry

**Table 3:** Sample for the focus group interview

Participant pseudonym	Professional role
Viv (BSc)	Band 6 district nurse
Yvonne (BSc)	Band 6 district nurse
Steve (MSc)	Band 7 High-Risk podiatrist and NHS podiatry team leader
Alexa (BSc)	Pre-practice podiatry graduate
Zena (BSc)	Pre-practice podiatry graduate
Trevor (BSc)	NHS podiatry services manager
Warren (MSc, PhD)	Professional association representative with history in high-risk podiatry specialism
Ursula (MSc, PhD)	Vascular Nurse Specialist and Academic

### Data analysis

Observation notes and audio files were transcribed verbatim. Framework analysis was used following the structured process of; familiarisation, identifying a thematic framework, indexing, charting, mapping, and interpretation suggested by Spencer et al. [49]. Framework analysis was chosen due to its matrix structure allowing large amounts of data from multiple sources to be viewed and considered simultaneously. Data were managed using Microsoft Excel spreadsheets wherein observational, interview and focus group data could be considered together allowing triangulation of findings. Data collection and analysis were approached reflexively with efforts taken to promote trustworthiness throughout. Upholding credibility, dependability, transferability and confirmability during the process was given priority [48]. The researcher maintained a reflexive diary to accompany the analysis process in order to recognise potential areas of bias. Analysis was conducted by the lead author supported by monthly discussions with co-authors where themes were explored and developed. This iterative process allowed for the development of the final thematic framework.

## Results

Table 4

**Table 4:** A thematic framework to represent the results of analysis

**Main Themes**	**Current practice**	**Identity**	**Time**
**Sub themes**	Talking the talk Venous disease is not in the podiatry veins	Foot focussed Life and sole Inter-professional identity Priorities	Constraint or opportunity? Time is routine
**Main Themes**	**Autonomy**	**Education**	**Venous disease in health care**
**Sub themes**	Money is power Follow the guidelines	Undergraduate education Waiting for champions Theory–practice mis-match	Who does what and when

### Current practice

Throughout the interviews, podiatrists provided accounts of their approach to venous disease identification and prevention stating that this was part of their practice. Participants were comfortable sharing their practice of identification and assessment:-

We don’t do any tests for that as such but if they’ve got bad skin or venous changes I still give them advice, (Georgina, NHS podiatrist)

I mean obviously if there’s a potential problem developing then (I) usually give advice, even things like maybe suggest they ask about the support stockings, things like that (Alice, private podiatrist).

However, it was significant that pre-practice graduates reported minimal attention to venous disease from their placement experience. Naomi stated:

I think from the advice that I’ve heard, like put your legs up, but no, nothing that’s structured that I could take away and think “oh that, that were really good” (Naomi, pre-practice podiatry graduate).

This corroborated recurrent themes in observation notes when podiatrists did not routinely or overtly assess or examine those patients who were at risk of venous disease or with signs of the condition. The data suggested a lack of knowledge and confidence in performing venous disease assessment and management.:-

Beryl palpated the pulses of patients whilst asking about current foot problems and preparing to start treatment. For many patients there were signs of venous disease in the form of telangiectasia and oedema yet this was not commented on or apparently investigated any further. (Beryl, private podiatrist, observation notes).

Alongside the mis-match between stated action and observed action participants recognised that lower limb venous disease was deserving of more than its peripheral presence in practice. It was not seen as core podiatry or a priority despite this recognition:-

They’ve [podiatrists] done a fantastic movement in the line of solving, or helping to solve a problem of peripheral arterial disease. Well that’s tiny numbers compared to venous disease and soft oedema. (Ursula, vascular nurse specialist and academic, focus group).

This is the sleeping giant and actually proportionately, it is probably a major amount of our patients, but it’s not being reviewed, looked at or otherwise. (Martin, podiatry academic).

Indeed, across the professions included in the focus group and the podiatry participants there was a lack of clarity as to whose role it was to identify and prevent lower limb venous disease:-

I would ask the nurse to do the venous assessment, but I don’t even know if that’s their role, I’m not sure on that one. It’s a grey area. Hmm. I’m not sure. (Donna, private podiatrist).

…we’ve got a problem when you get to management but we’ve got a bigger problem in terms of awareness and prevention… nobody treats soft oedema of a lower leg, everybody goes, “ooh that’s nice and soft isn’t it?” and walks away from it and waits for it to ulcerate. (Ursula, vascular nurse specialist and academic).

### Identity

All participants agreed that prioritisation of the foot was as a significant feature of the podiatrist’s role and identity. This identity often led to a lack of attention to any structure above the foot and evidence of a poor understanding of lower limb venous disease. Participants recognised that focussing on the feet did limit practice; it portrayed an identity to other professionals of a restricted scope and therefore a reduced opportunity for involvement further up the limb. Paul and Yvonne stated:

…it seems to be as well that other people leave it to podiatrists, other healthcare professionals, if it’s anything below the knee, even though we cover further up but, “oh podiatrist’ll sort that out” (Paul, pre-practice podiatry graduate).

…we just associate podiatrists with feet, we don’t see what other skills you’ve got. (Yvonne, district nurse)

Lower limb venous disease was not aligned with the foot focussed identity, it was perceived as a nursing responsibility and not part of the podiatry role. Georgina and Rachel explain:

…sometimes if they needed some stockings or anything, I might tell them to see their nurse. So I still look at venous, and still be aware of them. (Georgina, NHS podiatrist).

…but it’s like when you’re faced with a patient with venous problems, I think, other than observational signs, and referrals, what are we doing? (Rachel, podiatry academic).

The narrow clinical focus in practice inhibited podiatrists’ attention to the identification and prevention of venous disease. However, a contradiction to the foot focussed identity occurred when participants described the multi-factorial nature of their role and identity. Participants frequently adopted roles beyond that of an allied health professional by extending into psychological, social care or even social worker roles. Beryl described herself as being a point of human contact and listening service for patients stating:

(I am) The counsellor (Beryl, private podiatrist)

Indeed, participants from both private practice and the NHS provided similar accounts depicting two distinct areas of identity: the foot focus and the wider caring role:

In between appointments Fran told me, "the other nice thing in private practice is that patients come for a chat and are coming for counselling really." (Fran, private podiatrist, observation notes).

Sometimes some patients come in and pour everything out to us and you do end up talking more about different things (Georgina, NHS podiatrist)

### Time

Participants’ accounts suggested that practice was restricted by insufficient time with limited appointment durations that did not allow for all required clinical work to be completed.

What I found when I worked in the NHS was I never really looked above the knee. I never looked at them [Patients]… I never really took any notice, because I didn’t care, I didn’t have enough time to care. I was just doing my job. (Cathy, private podiatrist).

However, many participants, especially those employed in the private sector suggested the duration of podiatry appointments was an opportunity to address wider health issues and promote general wellbeing.

… it’s probably three times longer in time than a GP consultation that a podiatrist gets with a patient, so the opportunity to discuss your long-term conditions, your actual general medical health that impact on your foot health is a great opportunity but I don’t think it’s used at all. (Fran, private podiatrist).

This was evidently the case for some high priority conditions such as diabetes and peripheral arterial disease, but not for venous disease. Where time constraints existed, it was apparent that venous disease investigation was not prioritised and so did not feature in participants’ clinical routines. Instead, the focus on physical treatment took precedence.

I think the podiatrist’s got a big role to play in terms of health promotion and education and I don’t think we do that, I think the patient sits down, people pick clippers up and cut their nails even if they don’t need doing. (Fran, private podiatrist).

### Autonomy

Funding of appointments was discussed throughout data collection as an influence over clinical activity. A persistent theme emerged that payment for specific practice drove podiatrists’ use of their time and restricted their opportunities to pursue a holistic approach to patient care. Practice was not unsafe but often the source of funding appeared to influence clinical decisions as to what actions to take. A podiatrists’ appointment time was a commodity and functions performed in that time were determined by the source of funding.

I think they feel that they’re paying so they come in and they’ll tell you what they want and you’ll do what they want (Fran, private podiatrist)

Because ultimately we’re paid to do things that the Commissioners tell us to do, either via a block contract or an add-on, and if it lies outside of that we don’t get paid, and I suppose that, that in itself is a big driver… (Kate, NHS podiatry services manager).

Clinical guidelines were identified as a further threat to autonomy. Clinicians recognised the value of consistent delivery but also a feeling that these were an imposition rather than a choice.

The diabetes pathway is promoted strongly by NICE (National Institute for Health and Care Excellence) and there’s so much to go wrong that deviating from that pathway, you are putting your future career at risk (Leonard, NHS podiatry services manager and academic).

Is it not all NICE guidelines and stuff like that, we have to meet NICE guidance with regard to certain side of our profession and certain things have been thrust upon us…(India, NHS podiatrist).

Indeed, the feeling was that some of the lack of venous activity was due to absence of a specific set of guidelines and pathways:-

(there is a) lack of pathway or an assessment or a pathway that would prompt you. We’ve got pathways for claudication, all the ischemic pathways, all the foot ulcer pathway, but there’s no venous escalation methods that I know of. (Donna, private podiatrist).

I can see the benefit in doing that to prevent problems further down the line. It’s not there at the moment, and I think if we had enough evidence and I think if it came in a more directed way through our Commissioners then we probably would be going down that route. (Kate, NHS podiatry services manager).

### Education

Undergraduate education content and a lack of venous related leadership from within the profession emerged, through participant accounts, suggesting education was a limiting factor on practice. Conversely, throughout data collection the participants also recognised that their training included lower limb venous disease:

I did physiology within the system. Causes of venous problems, I got that. The usual sort of DVT, pregnancy, that sort of thing. it wasn’t a huge part of the syllabus, it was in there…(Eddie, private podiatrist).

However, views also emerged suggesting the undergraduate syllabus did not provide podiatrists with the skills and confidence to diagnose and manage many vascular pathologies including intermittent claudication and peripheral venous disease.

I think they [students] lack the ability to have a good general medical knowledge and I think that sometimes that can affect our profession. That’s where the, “oh you only cut nails”, comes from or the view or the vision of what a podiatrist does and I think there should be more core medical training as an undergraduate. (Fran, private podiatrist).

How they train students now is different to how they trained students when I was training which was thirty years ago this year and I do think students tend to kind of cut off at the feet and not think much more than the feet sometimes, they don’t think about the patient…(Heather, NHS podiatrist).

Questions were also raised as to the responsibility of podiatrists to engage in ongoing education and the availability of literature and resources:-

You need the right champions pushing it forward, you need the right enthused person to keep pushing it. (Steve, high risk specialist podiatrist and NHS podiatry team leader, focus group).

So maybe it’s because I’ve not read around it, I’ve not ever seen anything as an article about venous supply and the complications of it so it’s not ignited my imagination much (Beryl, private podiatrist).

Moreover, data indicated a theory–practice gap whereby the undergraduate acquisition of skills and knowledge had not persisted into practice. Several opinions arose to explain this:-

It was covered but I think then because notoriously…it’s like you don’t use it you lose it…. We have actually put an update on, on peripheral arterial disease and we were questioned on the veins and we all struggled as a department. We could all label all the arteries but not the veins (Donna, private podiatrist).

## Discussion

To the best of our knowledge this is the first study to explore the role of UK podiatry in recognising and preventing lower limb venous disease. The results of our ethnographic exploration have demonstrated a lack of clarity in the role. Combining multiple data collection methods revealed contrasts between spoken accounts and observed actions. These contrasts have brought to the fore important issues of professionalism, identity, culture and use of time with patients. Many of the findings are real world examples of discussion points raised in the Saks report [43] regarding the UK podiatry workforce development, and expose an opportunity for the profession to extend confidently into preventative practice beyond the foot. The findings are clear that participants recognised the opportunity to examine patients’ legs for signs of venous deterioration. This was in keeping with discursive literature claiming a role for nurses to identify venous changes and enact prevention opportunistically [1, 15–18, 20, 22–24]. However, there was an overriding participant perception that podiatrists prioritised their time for routine podiatry treatment.

The lack of a defined role for identification and prevention of venous disease is maintained by a strong identification as a foot focussed profession. Prioritisation towards patient comfort was a reason for podiatrists proportioning their time towards the physical treatment of the foot rather than health promotion and disease prevention. According to identity theories, podiatrists acting in accordance with the expectations of the role would feel secure, less distressed and therefore more likely to maintain strength of identity of the role [50–53]. Therefore, whilst there was evidence of their understanding of venous disease and its importance, it was not congruent with the main identity and clinical role. The findings highlight the need for podiatrists to embrace their role and identity in health promotion and public health. Such a role has been championed in the past and called for again in the recent Saks report [53–55, 27, 44]. However, the pressures of time, funding, and resulting clinical focus and culture mean this may still be a limited aspect of practice.

Cultures have been described to have ideal and real elements [56, 57]. Ideal culture consists of moral evaluations, by its members, of what behaviour ought to be in certain situations. Real culture conversely is the actual behaviour that occurs. Participants’ desire to act holistically and fulfil their duty of care to patients manifested in statements of ideal behaviour, however this was contradicted across interview data and during observations suggesting this had never been an element of real cultural behaviour. The contrast between ideal and real culture encapsulates the finding that podiatrists have an undefined and minimal role in identification and prevention of lower limb venous disease.

This was particularly evident for venous disease but data also showed a contrast with the preventative role participants took for other conditions. Diabetes and peripheral arterial disease both appeared more central to the work of podiatry than lower limb venous disease. Patients with diabetes are given priority, across healthcare in the UK, for amputation prevention. Ahmad et al. [58] highlighted that diabetes is not the sole cause of lower limb amputation and called for re-consideration of access to specialist foot protection services to incorporate peripheral arterial disease and other threats to limb viability. Guest et al. [59] used a retrospective cohort analysis of 505 patients with a diagnosis of venous leg ulceration and estimated the mean cost of wound care at £7600 per annum. Despite this, and partly due to baseline amputation data, the emphasis in podiatry prevention remains on diabetes and peripheral arterial disease. Focus on primary prevention is growing rather than established, reflecting that primary prevention has long been a subsidiary issue in the venous disease literature, guidance documents, and legislation [60].

Evidence from this study suggests more awareness is required, however the emergence of Legs Matter, the All-Party Parliamentary Group on vascular and venous disease and the Manchester Amputation Reduction Scheme (MARS) all suggest progress in a positive direction toward a culture of lower limb wound prevention across all health care. As lower limb specialists, podiatrists are implicated and involved in creating that culture but this has not happened yet in terms of lower limb venous disease across practice. On the evidence from this study, awareness campaigns, professional development training and policy changes would be required to instigate a shift of podiatry’s focus away from solely attending to foot pathology to truly embracing pathologies of the lower limb.

### Study limitations

This was a relatively small-scale study, limited to regional data collection. This might mean that other regions show pockets of practice that stand outside the findings of this study. However, measures were taken to ensure a broad, representative sample was selected in order to explore and reflect the realities of practice. Whilst a qualitative study would not claim generalisation [48], the sampling process and range of voices included suggests there is potential for podiatrists within the UK to recognise these findings as familiar in respect of their own practice and experience. Nevertheless, the authors acknowledge that the personal perspective of researchers in qualitative studies can influence decisions at all stages, although all attempts to overcome personal bias during the study were taken.

## Conclusion

Lower limb venous disease is both a consequence and contributor to the current health care crisis and more preventative action is required to diminish the effects of this devastating condition [3, 13, 14, 61]. Timely attention to prevent disease deterioration could limit the complications associated with lower limb venous disease and ultimately improve quality of life [8]. The findings from this study provide a foundation for future research and discussion within the profession surrounding how podiatrists might use their time for education and health promotion with at-risk patients to prevent deterioration in lower limb venous status. Further research could add to the contribution of this study, building on the new knowledge of podiatry’s current role in lower limb venous disease and the factors influencing it. The knowledge gained through this study suggests a gap exists for exploration of the impact of incorporating early identification and prevention into practice. As a profession, podiatrists are in a prime position to identify patients at risk of lower limb venous disease and work towards the reduction and prevention of it, thereby increasing quality of life for many individuals.

## Data Availability

Not applicable.
